# Hygrothermal stress increases malignant arrhythmias susceptibility by inhibiting the LKB1-AMPK-Cx43 pathway

**DOI:** 10.1038/s41598-024-55804-0

**Published:** 2024-02-29

**Authors:** Jianing Chi, Ningxia Wu, Pengfei Li, Jiaman Hu, Hua Cai, Cailong Lin, Yingying Lai, Han Yang, Jianyu Huang, Min Li, Lin Xu

**Affiliations:** 1 The First School of Clinical Medicine, Southern Medical University, Guangzhou, China; 2 Department of Geriatric Cardiology, General Hospital of Southern Theater Command, Guangzhou, China; 3 Branch of National Clinical Research Center for Geriatric Diseases, Chinese PLA General Hospital, Guangzhou, China; 4 Guangzhou Key Laboratory of Cardiac Rehabilitation, Guangzhou, China; 5 Graduate School, Guangzhou University of Chinese Medicine, Guangzhou, China; 6 School of Public Health, Guangdong Pharmaceutical University, Guangzhou, China

**Keywords:** Cardiology, Diseases

## Abstract

High mortality due to hygrothermal stress during heat waves is mostly linked to cardiovascular malfunction, the most serious of which are malignant arrhythmias. However, the mechanism associated with hygrothermal stress leading to malignant arrhythmias remains unclear. The energy metabolism regulated by liver kinase B1 (LKB1) and adenosine monophosphate-activated protein kinase (AMPK) and the electrical signaling based on gap junction protein, connexin43 (Cx43), plays important roles in the development of cardiac arrhythmias. In order to investigate whether hygrothermal stress induces arrhythmias via the LKB1-AMPK-Cx43 pathway, Sprague–Dawley rats were exposed to high temperature and humidity for constructing the hygrothermal stress model. A final choice of 40 °C and 85% humidity was made by pre-exploration based on different gradient environmental conditions with reference to arrhythmia event-inducing stability and risk of sudden death. Then, the incidence of arrhythmic events, as well as the expression, phosphorylation at Ser368, and distribution of Cx43 in the myocardium, were examined. Meanwhile, the adenosine monophosphate-activated protein kinase activator, Acadesine, was also administered to investigate the role played by AMPK in the process. Our results showed that hygrothermal stress induced malignant arrhythmias such as ventricular tachycardia, ventricular fibrillation, and severe atrioventricular block. Besides, hygrothermal stress decreased the phosphorylation of Cx43 at Ser368, induced proarrhythmic redistribution of Cx43 from polar to lateral sides of the cardiomyocytes, and also caused LKB1 and phosphorylated-AMPK expression to be less abundant. While, pretreatment with Acadesine significantly actived the LKB1-AMPK-Cx43 pathway and thus ameliorated malignant arrhythmias, indicating that the hygrothermal stress-induced arrhythmias is associated with the redistribution of gap junctions in cardiomyocytes and the organism's energy metabolism.

## Introduction

Extremely high temperatures and humidity, as a severe stress element, will most likely have negative implications as global climates change. Acute hygrothermal stress (HHS) can result in heat stroke (HS), a deadly pyrogenic condition with a morbidity and fatality rate as high as 40%^1^. Over the past several decades, the prevalence of HS has drastically grown; during the summer heat waves, the incidence ranges from 17.6 to 26.5 per 100,000 people, and up to 60% of patients with characteristic HS are hospitalized or discovered dead within one day of the reported beginning of illness^2,3^. Furthermore, it is predicted that by 2030, the economic costs of heat stroke would surpass $2.4 billion^4^. The cardiovascular system is crucial to the systemic heat response and organ perfusion during sudden death brought on by HHS^5^. The percentage of cardiovascular dysfunction in individuals with severe HS that had multiple organ failure can range from 43.4 to 65.2%^6^. Up to 90% of the overall increase in mortality during heat waves has been linked mostly to the cardiovascular system, with malignant arrhythmias serving as the primary reason^7^. Arrhythmias do occur during HHS, but the underlying pathophysiologic and molecular processes are still poorly understood, and there aren't many effective and focused treatment options. It is vital to investigate the best course of action for its successful treatment to lower mortality.

Gap junctions, a unique membrane structure made of connexin (Cx), are the structural basis for electrical, metabolic, and mechanical coupling between neighboring cells. They are also an important channel for electrochemical signaling, maintenance of inter-cardiac communication, regulation, and assurance of normal cardiac rhythmicity by neighboring cardiomyocytes, which plays a crucial role in the development of cardiac malignant arrhythmias^8^. Cx43, the primary channel protein produced by cardiac working cells, is exclusively present in the atrial and ventricular myocardium as well as the distal conduction system of all adult mammalian hearts. It is located primarily in the intercalated disc and is essential for the electrical coupling of cardiomyocytes, which ensures action potential and molecular signal propagation in the heart^9,10^. While the disruption caused by changes in the expression and topology of Cx43 under stress conditions is a key factor in arrhythmogenesis and even the occurrence of sudden cardiac death^11–13^.

Arrhythmia development and improper energy metabolism in cardiomyocytes are intimately connected^14^. Adenosine monophosphate-activated protein kinase (AMPK), a serine-threonine kinase, controls energy metabolism as well as other cellular functions such as autophagy, apoptosis, oxidative stress, and inflammation^15,16^. Its activity has a significant role in a number of cardiovascular illnesses that cause arrhythmias by downregulating ion channels and gap junctions, changing arrhythmogenic circumstances, and causing electrophysiological dysfunction^17,18^. In addition, it has been shown that activation of AMPK is involved in the preservation of Cx43 and the reduction of reactive oxygen species^19,20^. This effect may be related to the amelioration of inflammatory response and oxidative stress due to hygrothermal stress, which further reduces the occurrence of arrhythmias^20,21^. Meanwhile, as a key upstream kinase of AMPK, liver kinase B1 (LKB1) phosphorylates and activates AMPK to control cellular functions and energy metabolism^22^. However it has not yet been determined whether HHS causes arrhythmias by controlling Cx43 expression via the LKB1/AMPK pathway.

In this study, we investigated whether hygrothermal stress influences the myocardial remodeling of Cx43 via the LKB1/AMPK pathway, leading to an increased susceptibility to malignant arrhythmias. We substantiated our hypothesis by constructing a rat model that simulates malignant arrhythmias induced by hygrothermal stress. Utilizing this model, we demonstrated that pretreatment with the AMPK agonist AICAR can ameliorate the remodeling of Cx43, thereby exhibiting antiarrhythmic effects.

## Results

### The progression of Tr and HR under HHS is slowed down by activating AMPK

**Figure 1:**
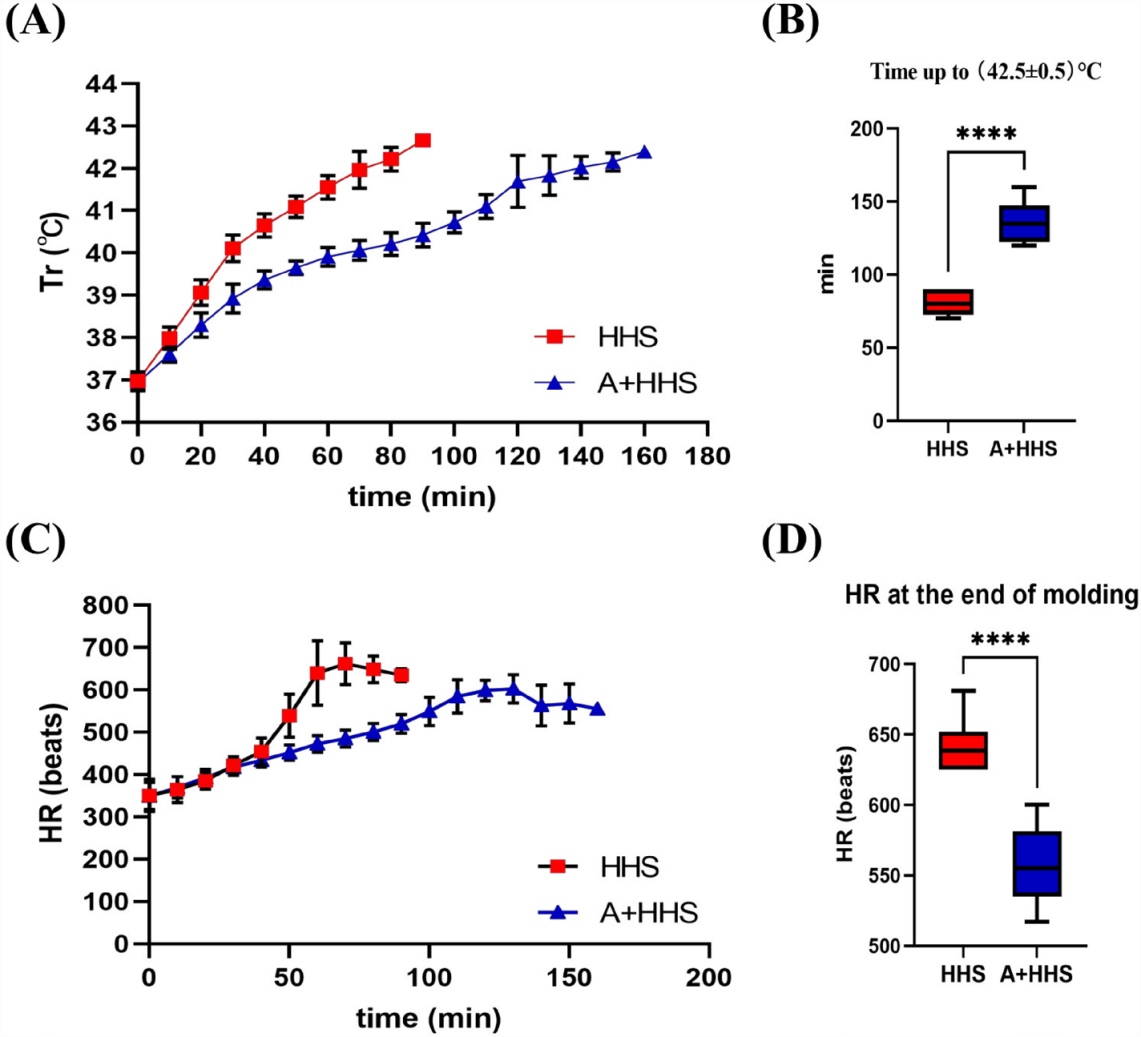
Tr and HR trends under HHS are impacted by AMPK activation. (**A**) Tr trends through time for each group of rats. (**B**) The time for Tr to reach (42.5 ± 0.5)°C was significantly longer in AICAR pretreated rats than in the HHS group (n = 8). ****P < 0.0001. (**C**) HR trends over time during hygrothermal exposure. (**D**) HR at the end of modeling was significantly lower in the A + HHS group than in the HHS (n = 8). ****P < 0.0001.

After entering the HHS chamber, the Tr of rats climbed quickly from the Tcb to 40 °C within 30 min, and the rate of increase of Tr reduced in the following 20 min before reaching (42.5 ± 0.5) °C in about 80 min (Fig. 1A). In the meantime, the HR in rats was practically constant for 30 min from the start of the hygrothermal exposure, then climbed quickly within 30 min, peaking at (41.8 ± 0.15) °C, and then fell slightly until the modeling was complete (Fig. 1C). Yet, early treatment with an AMPK activator significantly prolonged the mean time for Tr to reach (42.5 ± 0.5) °C in HHS-Exposed rats from 81.25 to 136.25 min (Fig. 1B). Besides, AICAR also markedly reduces the development of HR in rats under HHS (Fig. 1D).

### Activation of the AMPK by the AICAR injection attenuates arrhythmias in rats

**Figure 2:**
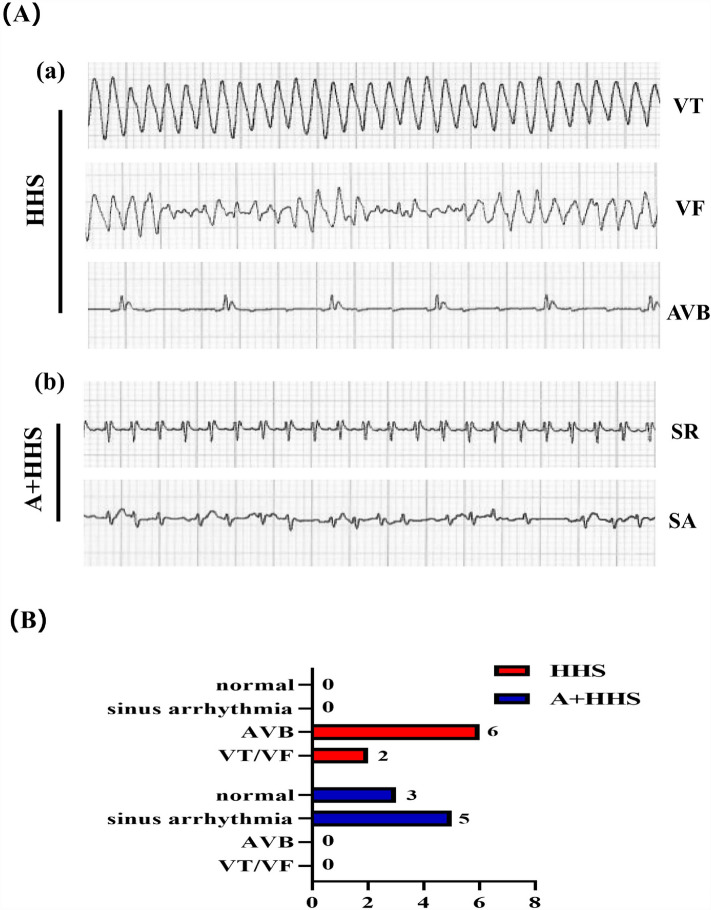
HHS causes malignant arrhythmias, whereas AICAR lowers the occurrence and severity of arrhythmias. (**A**) Typical ECG recordings for HHS or A + HHS. (a) The main types of malignant arrhythmias seen in the HHS group were ventricular tachycardia, ventricular fibrillation, and high degree of AV block; (b) After AICAR pretreatment, the A + HHS group did not exhibit malignant arrhythmias, only showing sinus rhythm abnormalities. (**B**) Number of rats with arrhythmias in each group (n = 8).

Due to physiological changes such as electrolyte imbalances and dehydration, sinus arrhythmic events such as sinus tachycardia can happen in high temperature and high humidity environments. In the current study, we discovered an increased propensity for extreme HHS to cause fatal arrhythmias such as ventricular tachycardia (VT), ventricular flutter (VF), and high/third-degree atrioventricular block (AVB) (Fig. 2A.a). Additionally, we demonstrated that pre-injecting rats with AICAR dramatically reduced the occurrence of ventricular arrhythmias during HHS compared to untreated rats and that HHS also decreased the susceptibility of rats to AVB (Fig. 2A.b,B).

### Hormonal stress markers and inflammatory factors were both upregulated by HHS

**Figure 3:**
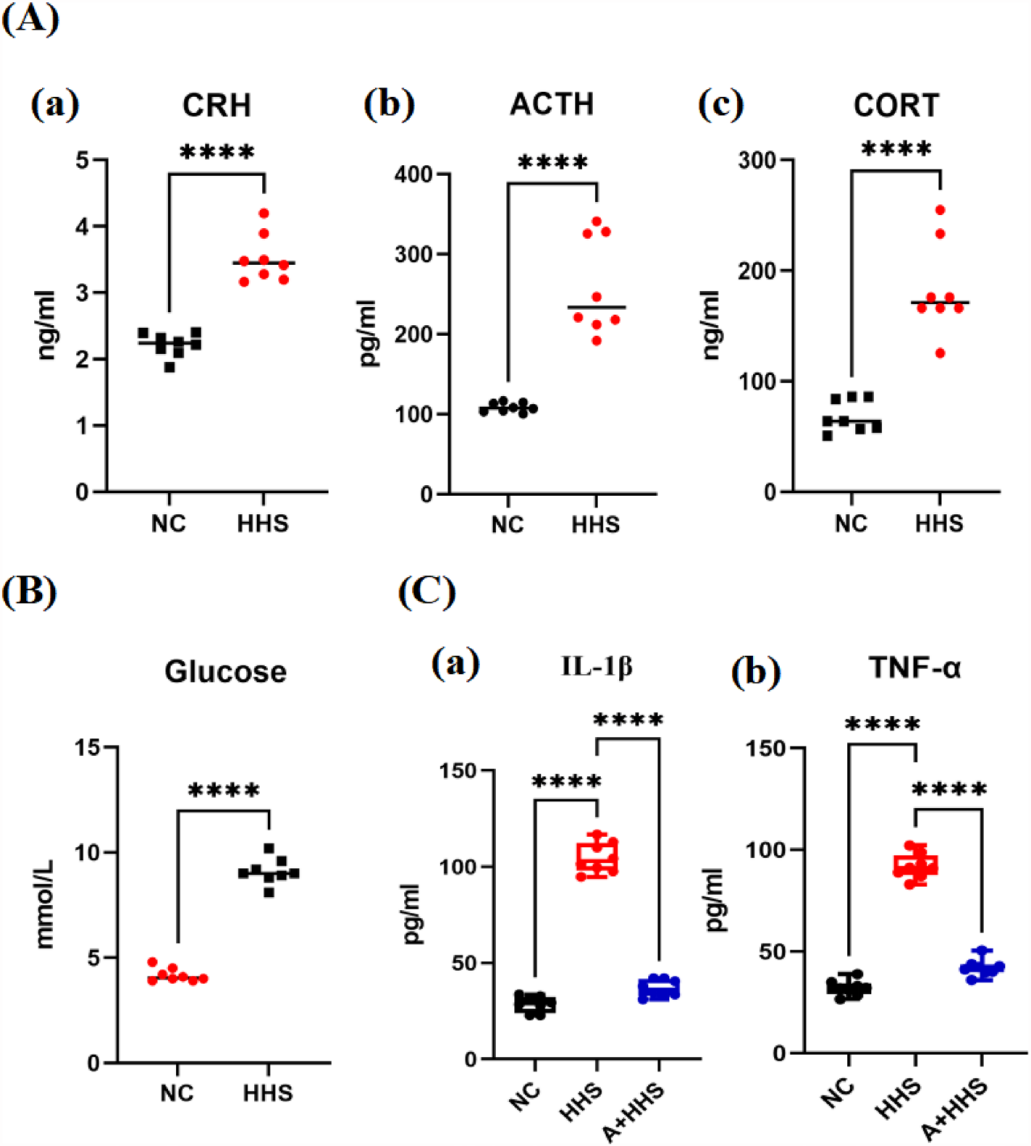
Hygrothermal exposure causes acute stress in rats and raises levels of inflammation. (**A**) Elisa test was used to quantify the stress hormones. (a–c) CRH, ACTH and CORT were significantly elevated in serum of rats after HHS. (**B**) HHS significantly increased blood glucose levels in rats. (**C**) Elisa test for serum inflammatory factors. (a, b) Expression of IL-1β and TNF-α were significantly increased by HHS exposure, but levels decreased markedly after AICAR pretreatment.****P < 0.0001.

Following the end of the hygrothermal exposure, the serum expression levels of the hormone markers ACTH, CRH, CORT, and blood glucose were considerably higher in the HHS group than in the NC (Fig. 3A,B). In addition, HHS had higher levels of IL-1β and TNF-α, which dramatically decreased after receiving AICAR (Fig. 3C).

### HHS remodeled Cx43 by inhibiting activation of the LKB1/AMPK pathway

**Figure 4:**
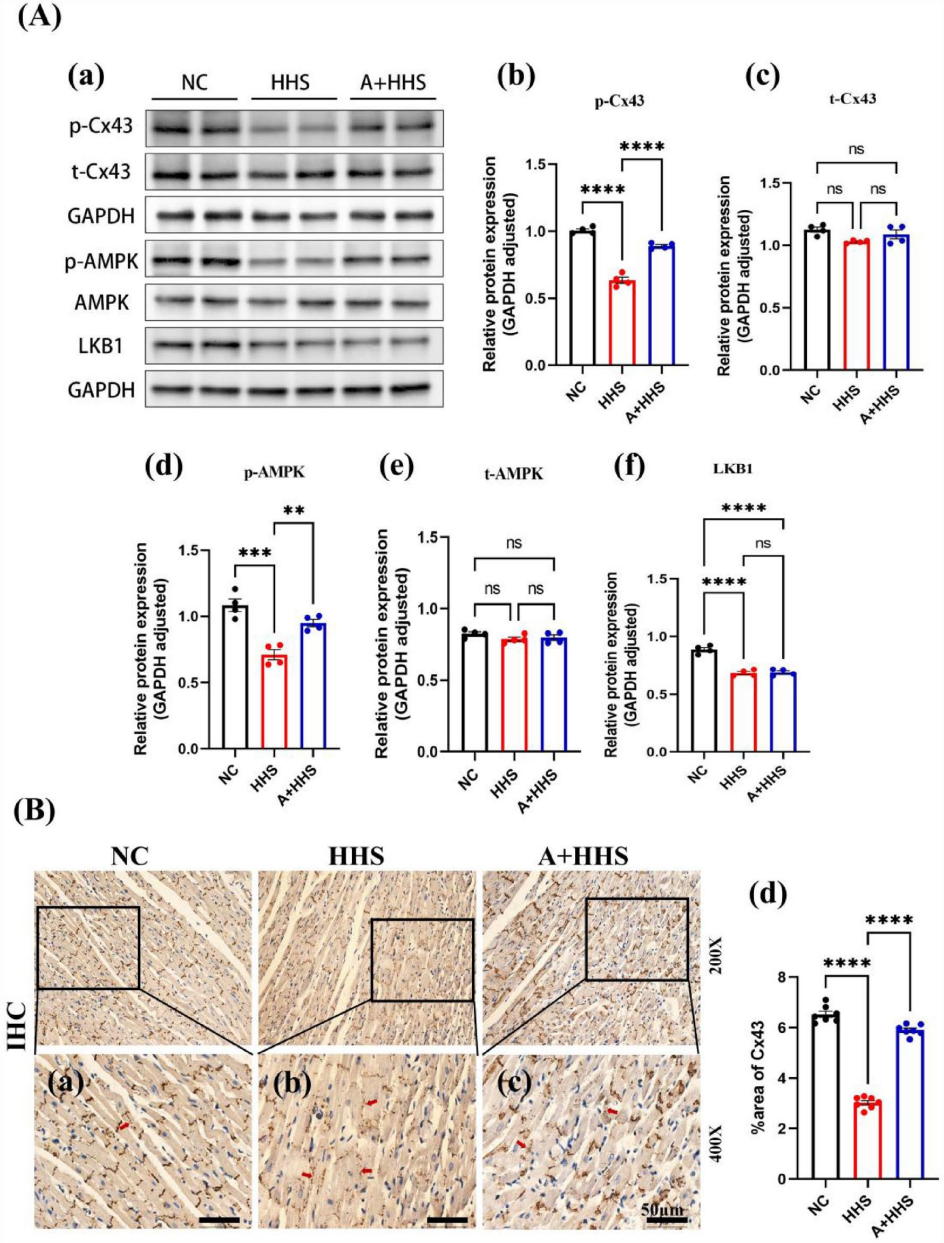
HHS affects Cx43-pS368 expression and distribution via the LKB1/AMPK pathway. (**A**) Results of western blot. (a) Western Blot demonstration of p-Cx43, t-Cx43, p-AMPK, t-AMPK, and LKB1 in cardiac tissues from NC, HHS, and A + HHS. (b–f) Quantification of the p-Cx43, t-Cx43, p-AMPK, t-AMPK, and LKB1 level (n = 4). (**B**) IHC staining of Cx43-pS368. (a) Normal Cx43-pS368 expression in the NC group was high and basically distributed in the transverse junctions, i.e., "end-to-end"; (b) Cx43-pS368 expression was reduced and changed to "side-to-side" distribution in the HHS group; (c) Improvement in the expression and distribution of Cx43-pS368 in the A + HHS group after pretreatment with AICAR; (d) Quantification of the positive area in myocardial tissues from three groups (n = 7). Scale bar = 50 μm. ns *P* > 0.05, *P < 0.05, **P < 0.01, ***P < 0.001, ****P < 0.0001.

The expression and phosphorylation at Ser368 levels of the proteins Cx43 and AMPK during HHS were revealed by the western blot data. When compared to the NC group, the HHS group showed significant downregulation of p-Cx43 (Ser368), p-AMPK (Thr172), and LKB1 expression (Fig. 4A.a–f). Following pretreatment with AICAR, the expression of Cx43-pS368, and p-AMPK (Thr172) were significantly higher in the A + HHS group (Fig. 4A.b–d). Nevertheless, AMPK activation does no effect on LKB1 expression (Fig. 4A.f), and there was no statistically significant difference in total AMPK across the three groups (Fig. 4A.e). Meanwhile, immunohistochemical (IHC) staining showed that the distribution of Cx43-pS368 in NC was centered on the intercalated discs (end-to-end junctions of cardiomyocytes) of cardiomyocytes, with a linear distribution perpendicular to the longitudinal axis of cardiomyocytes (Fig. 4B.a). In contrast to NC, the Cx43-pS368-positive area in HHS myocardial tissues was greatly decreased (Fig. 4B.d), and the distribution of myocardial regional staining spots and plaques was random and primarily dispersed in the shape of side-to-side junctions (Fig. 4B.b). After receiving AICAR pretreatment in the A + HHS group, Cx43-pS368 expression and its "end-to-end" distribution ratio considerably increased when compared to HHS (Fig. 4B.c and d).

### HHS leads to myocardial injury and fibrosis

**Figure 5:**
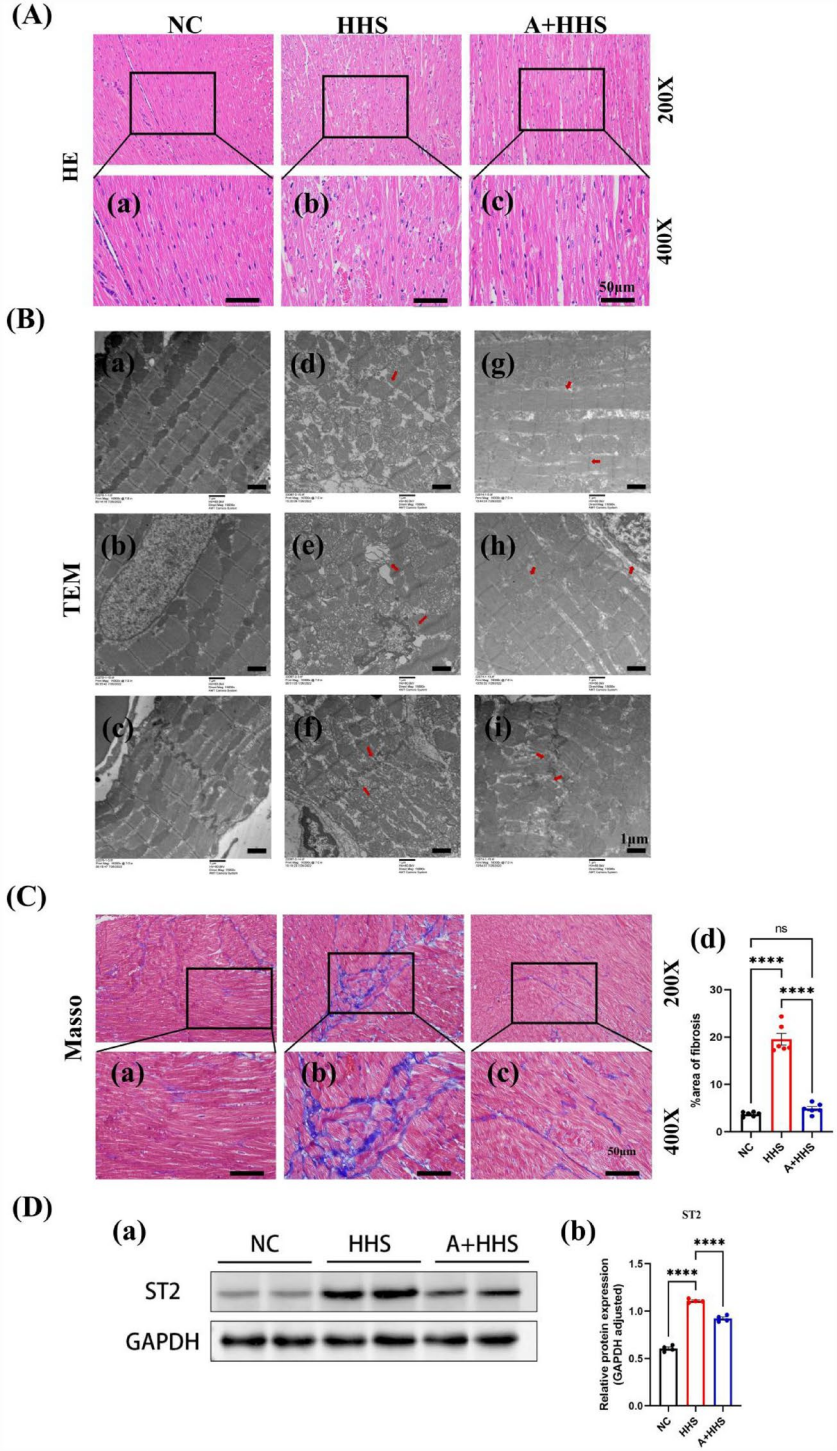
Activation of the AMPK by AICAR injection inhibits HHS-induced myocardial injury and fibrosis. (**A**) Representative HE staining results of rat myocardial tissues in all groups. (a) Normal expression of myocardial structure of NC group; (b) Alterations in Myocardial Structure in the HHS Group; (c) Improvements in myocardial structure in the A + HHS group. (**B**) Ultrastructural deformation of rat cardiomyocytes in each group was shown by TEM imaging. (a–c) Normal ultrastructure of the NC group; (d–f) Ultrastructural alterations in the HHS group; (g–i) Improvements of ultrastructure of A + HHS group. (**C**) Typical Masson staining for fibrosis that stained in blue. (a) Normal Masson staining manifestation in NC group; (b) Significant fibrotic changes in HHS group; (c) Improvement of fibrosis in A + HHS group; (d) Quantification of the fibrotic area of the Masson-stained sections (n = 6). (**D**) Western Blot Analysis of ST2, a Marker of Myocardial Fibrosis. (a) Western Blot demonstration of ST2; (b) Quantification of the ST2 level (n = 4). ****P < 0.0001.

H&E showed that the transverse stripe of cardiomyocytes in the NC group was clear, the nuclei of the cells were dense, the staining was homogeneous, and the pathological manifestations, such as the inflammatory cellular infiltration, were not seen (Fig. 5A.a); whereas, in the HHS group, the myocardial tissue structure was blurred, cell gaps were enlarged, myofibrils were loosely arranged and disorganized, and some myofibrils were fractured and cleaved, with focal inflammatory cell infiltration and erythrocyte exudation (Fig. 5A.b). Additionally, TEM analysis revealed that myocardial myogenic fibers in the NC group exhibited a well-organized alignment, characterized by regular Z lines and distinct myofilaments. Intercalated disks were prominently visible and predominantly stepped in nature. Additionally, mitochondria and nuclei exhibited a normal and structured morphology (Fig. 5B.a–c). Conversely, a notable alteration in the ultrastructure of rat cardiomyocytes was observed following HHS exposure. Fiber gaps were widened, with some fibers even displaying signs of breakage, and the Z line appeared blurred. Intercalated discs were not clearly discernible, and the connecting gaps were widened. Mitochondria exhibited swelling, broken cristae, and vacuolation, indicative of structural damage. Furthermore, nuclei showed signs of atrophy, accompanied by irregular and jagged nuclear membranes (Fig. 5B.d–f). At the same time, Masson discovered that the HHS rats' cardiac tissue had a much higher level of fibrosis than the NC rats did (Fig. 5C.a,b and d). To further validate the extent of fibrosis due to HHS, we performed Western Blot analysis of ST2, a marker of myocardial fibrosis, showing the same results (Fig. 5D). Compared with the HHS group, the myocardial injury and fibrosis were significantly improved in the A + HHS group (Fig. 5A.c, B.g–i, and C.b–d).

## Discussion

**Figure 6:**
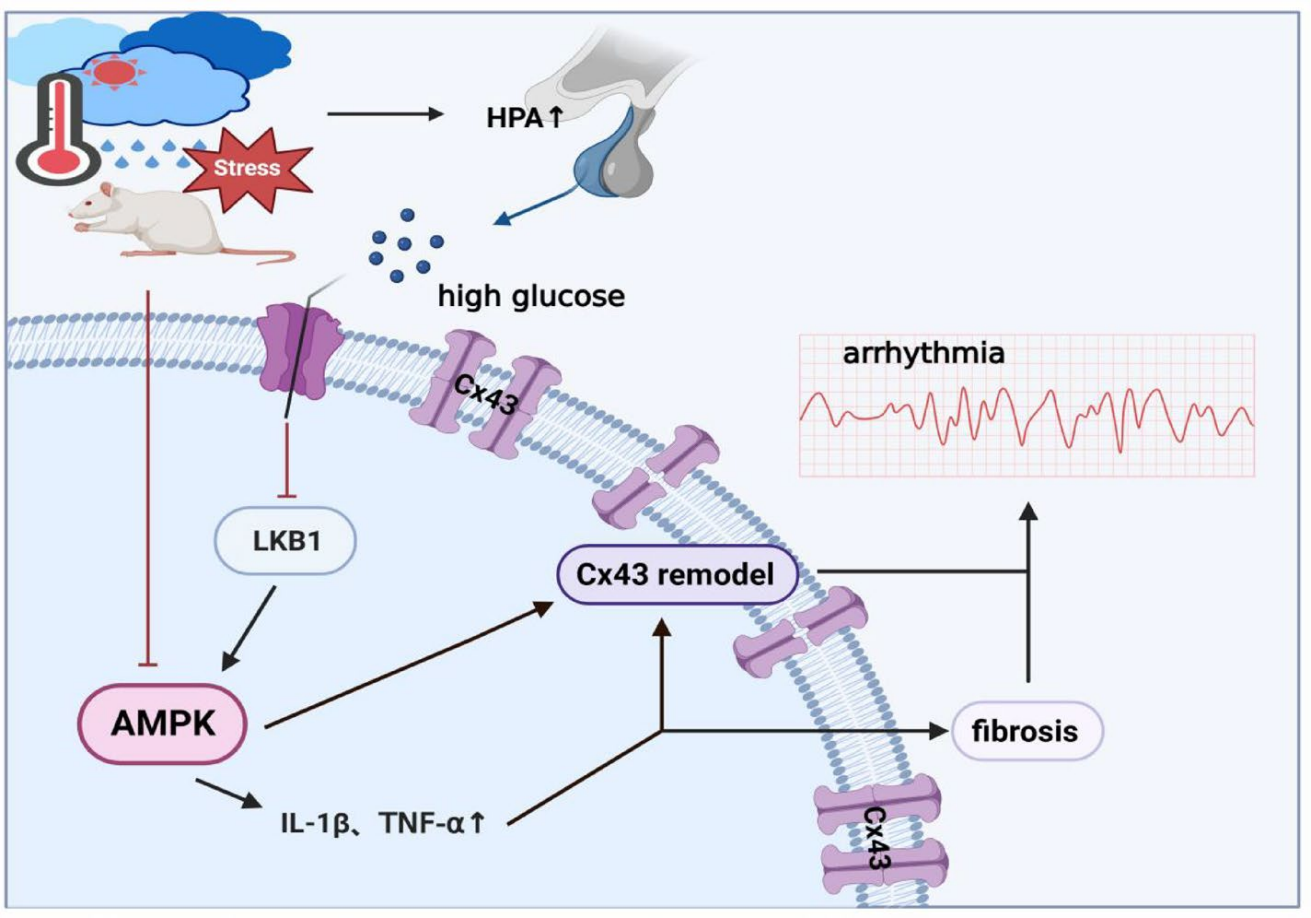
The possible process through which HHS modifies Cx43 and causes arrhythmia is depicted in a cartoon. By triggering the HPA axis, HHS raises blood glucose levels and suppresses the activity of LKB1. This, in turn, hampers the expression of AMPK, or HHS directly inhibits AMPK expression. Ultimately, these mechanisms contribute to the development of arrhythmias by further influencing the remodeling of Cx43 and initiating fibrosis after inflammatory responses.

In the current work, we provided the first evidence of the function of Cx43 in controlling the development of arrhythmias, inflammation, and cardiac fibrosis brought on by HHS. The cardiac tissues of post-HHS arrhythmic rats showed substantially reduced Cx43-pS368 expression as well as disorganized redistribution. Functionally, HHS caused cardiac fibrosis and inflammation, which made rats more vulnerable to dangerous arrhythmias. The HHS-induced index alterations were greatly mitigated by AMPK activation. From a mechanical standpoint, we showed that HHS prevented the LKB1/AMPK/Cx43 signaling pathway from being activated (Fig. 6).

Stress is defined as a sequence of physiological reactions initiated when the organism is aroused by numerous stressors in the internal and external surroundings, as well as several consequent changes in function and metabolism. The rhythm of the heart is impacted by stress, and in extreme circumstances, this can result in malignant arrhythmias or even abrupt cardiac death^23,24^. According to Benito B^25^, high temperatures and humidity levels might result in oxidative stress, which can compromise heart function and trigger cardiomyocyte death. Acute stress was created using a model by Liu et al.^26^ in goats, and it was shown that this stress resulted in a large rise in ventricular arrhythmias and altered ventricular electrophysiology. As a stressful disease that gravely jeopardizes life and health in the face of global warming, HS urgently requires the exploration of appropriate intervention targets and therapeutic strategies^27,28^. The effects of humidity were disregarded in earlier proposed animal models of HS^29–31^, which established desert-like environments. However, research has shown that people feel hotter in high humidity than in low humidity^32^. For this reason, in this investigation, we consulted methodologies from the literature and took into account the impact of humidity before deciding on 40 °C and 85% humidity as the ambient parameters for HHS-induced arrhythmias. What is interesting is that, in addition to the previously described VT and VF, rats were also particularly prone to AVB under this stressful environment. We hypothesize that the cause of this might be that abnormal ATP release during the HHS state results in increased secretion of inflammatory substances like IL-1β and TNF-α, which facilitates the growth of myocardial fibrosis and, in turn, causes abnormal atrial impulse formation and conduction, which, in turn, triggers the emergence of AVB^33,34^.

Gap junctions are implicated in the emergence of cardiac disorders such as arrhythmias, heart failure, and hypertrophy, according to mounting evidence^35,36^. Gap junction protein Cx43, the major isoform expressed in ventricular myocytes, is crucial in defining cardiac excitation patterns and arrhythmia susceptibility^37^. In various types of stress models, the reduced expression and phosphorylation at Ser368 of Cx43 decreases myocardial excitability and conductivity, alters the conduction of electrical impulses, and increases vulnerability to ventricular arrhythmias^38,39^. In our research, we found that the HHS-induced reduction in Cx43-pS368 suggest that Cx43 may potentially be implicated in the occurrence of various arrhythmias, including AVB and VT/VF. Cx43, the primary channel protein expressed by myocardial working cells, is primarily found in the intramyocardial disc in the form of "end-to-end," ensuring the directionality of electrical impulse conduction. A small amount of it is also found in the connection between the cardiomyocytes' two sides, or the "side-to-side" connection, coordinating the transmission of electrical signals between adjacent parallel myocardium and preserving continuity and synchronicity. Slower conduction and higher electric heterogeneity, which eventually served as the foundation for the development and maintenance of VAs, were induced by the downregulation of Cx43 and aberrant distribution brought on by HHS, particularly the increase in side-to-side connections. Moreover, downregulation of Cx43 and alterations in its distribution increase the dispersion and refractoriness of action potential duration and promote malignant arrhythmias such as early or delayed postdepolarization, conduction slowing, and conduction block^40^. In addition, Cxs, one of the less well-studied pathways, can also lead to fibrosis^41^. In studies of non-cardiac tissues, decreased Cx43 expression was associated with increased collagen deposition^42^. In the heart, Cx43 is predominantly expressed in cardiomyocytes and fibrosis, and its expression level correlates with the proliferative mechanism of fibroblasts^43^ and is further involved in arrhythmogenesis. We discovered that the dysregulation of cardiac gap junctions caused by Cx43-pS368 downregulation during the current HHS-induced arrhythmia correlated to increased fibrosis, which in turn further led to conduction anomalies and the genesis of conduction block. Furthermore, apart from the aforementioned factors, inflammation resulting from acute HHS, exemplified by a notable surge in IL-1β and TNF-α as observed in this experiment, along with oxidative stress, collectively impede the effective propagation of electrical signals between cardiomyocytes through the Cx43 gap junction channels. This inhibition contributes to electrical remodeling and destabilization of the heart, amplifying the occurrence of arrhythmias and the cardiac propensity for arrhythmias^44^.

We have further evidence that HHS changed the phosphorylation state of AMPK of the T172 site in the emergence of HHS-induced arrhythmias. We preinjected the rats with the AMPK agonist AICAR before the exposure in order to investigate the underlying mechanism of AMPK action in the development of HHS-induced arrhythmias. We discovered that Cx43 protein phosphorylation at Ser368 were noticeably increased in myocardial tissue, and the "side-to-side" distribution was diminished compared to the HHS group. Additionally, HHS-induced inflammation, ultrastructural abnormalities, and fibrosis are all greatly reduced by activating AMPK. Most importantly, AMPK activation markedly decreased vulnerability to HHS-induced arrhythmias. These findings, which are consistent with research by Alesutan^45^, suggest that AMPK regulates the Cx43-pS368 and the development of arrhythmia via HHS. HHS can trigger abnormal ATP release, whereas AMPK operates as an energy receptor in response to energy excess, and ATP competitively displaces AMP to decrease AMPK activity^46^. Remarkably, in response to heat stress, the body upregulates the expression of heat shock proteins (HSP) to enhance protective heat tolerance^47,48^. HSP is subject to negative regulation by AMPK and plays a crucial role in the stabilization of Cx43. However, our comprehensive review of existing literature and experimental findings consistently reveal that AMPK phosphorylation of the T172 site and Cx43-pS368 expression are suppressed during HHS. Consequently, the protective and anti-HHS effects of HSP on Cx43-pS368 may involve alternative pathways, necessitating further exploration through additional experiments. Moreover, recent discoveries^49,50^ have provided substantial evidence supporting the connection between the AMPK signaling system and Cx43 expression in cardiac fibroblasts, implying a potential role for this pathway in the common fibrotic response observed in various cardiac diseases. The decrease in AMPK phosphorylation of the T172 site enhances the production of pro-inflammatory molecules such as IL-1β and TNF-α, which mediate the inflammatory response, induce Cx43 remodeling, and further promote the formation of cardiac fibrosis for arrhythmogenesis^51^.

Furthermore, we showed that HHS influenced the protein expression of liver kinase B1 (LKB1), whereas LKB1 protein expression was not significantly altered after pretreatment with the AMPK pathway agonist. These findings suggest that in the process of HHS generating arrhythmias, liver kinase B1 (LKB1) works as an upstream master kinase to phosphorylate and activate AMPK, which impacts the apoptosis of cardiomyocytes and gap junctions^52^. Meanwhile, HHS considerably increased hypothalamus pituitary adrenal (HPA) hormone axis stress indexes. We hypothesize that this is because HHS stimulates the release of cortisol hormones by activating the HPA axis, which in turn activates the mitochondrial oxidative stress system, causing increased reactive oxygen species production, which leads to the dissociation of LKB1, reduces the interaction between LKB1 and AMPK, and decreases the level of phosphorylation of the T172 site, which inhibits AMPK activity^53^. AMPK activation may also protect cardiac gap junctions from remodeling in hyperglycemia, according to Li et al.^54^.

There are several constraints to our study. Although rodents are commonly employed in cardiac arrhythmia research, they are not appropriate animal models for this subject due to differences in heart size, heart rate, and action potential structure when compared to humans. Furthermore, we found that the decreased expression and aberrant distribution of Cx43 in HHS-induced arrhythmic rats were connected with AMPK, although we did not study the particular mechanism by which AMPK regulates Cx43-pS368. Therefore, it is unclear whether AMPK controls Cx43 remodeling via the "ubiquitin–proteasome system" or by noncatalytic processes such as protein complex folding, protein interactions, or subcellular targeting^55^. Furthermore, both LKB1 and Cammk2 operate as upstream kinases for AMPK, however, the involvement of Ca2 + in HHS-induced arrhythmias was not investigated in this work. These crucial concerns need to be carefully handled in the future.

As a conclusion, our recent research has shown that the genesis of arrhythmias is significantly influenced by the lower expression of Cx43-pS368 in the cardiac tissues of HHS rats. For the development of the arrhythmias brought on by HHS as well as the underlying fibrotic and inflammatory responses in the myocardium, Cx43 phosphorylation, and distributional remodeling are necessary. To the best of our knowledge, the current work is the first to demonstrate that acute HHS not only causes ventricular arrhythmias but also increases susceptibility to other malignant arrhythmias like AVB, and in the meanwhile we demonstrate the pathophysiologic relationship of Cx43 in this context. Our research suggests that activating AMPK inhibits the emergence of arrhythmias brought on by HHS and may represent a unique strategy for preventing arrhythmias by concurrently inhibiting inflammatory and fibrotic responses.

## Methods

### Ethics statement

All experimental protocols and methods were performed in accordance with the relevant guidelines and regulations by General Hospital of Southern Theater Command (IAC11e approval No. 104110701). And the portion of the study involving live animals was conducted in compliance with ARRIVE guidelines.

### Animals

We used 24 adult male Sprague–Dawley (SD) rats (280–350 g, GUANGDONG MEDICAL LABORATORY ANIMAL CENTER, Guangzhou, China) that were 8 weeks old. All experimental animals were housed under standard experimental conditions with a controlled ambient temperature of (22 ± 1) °C, relative humidity of (50 ± 5)%, a light–dark cycle of 12 h, and adequate feed and water sources.

### Experimental groups

**Figure 7:**
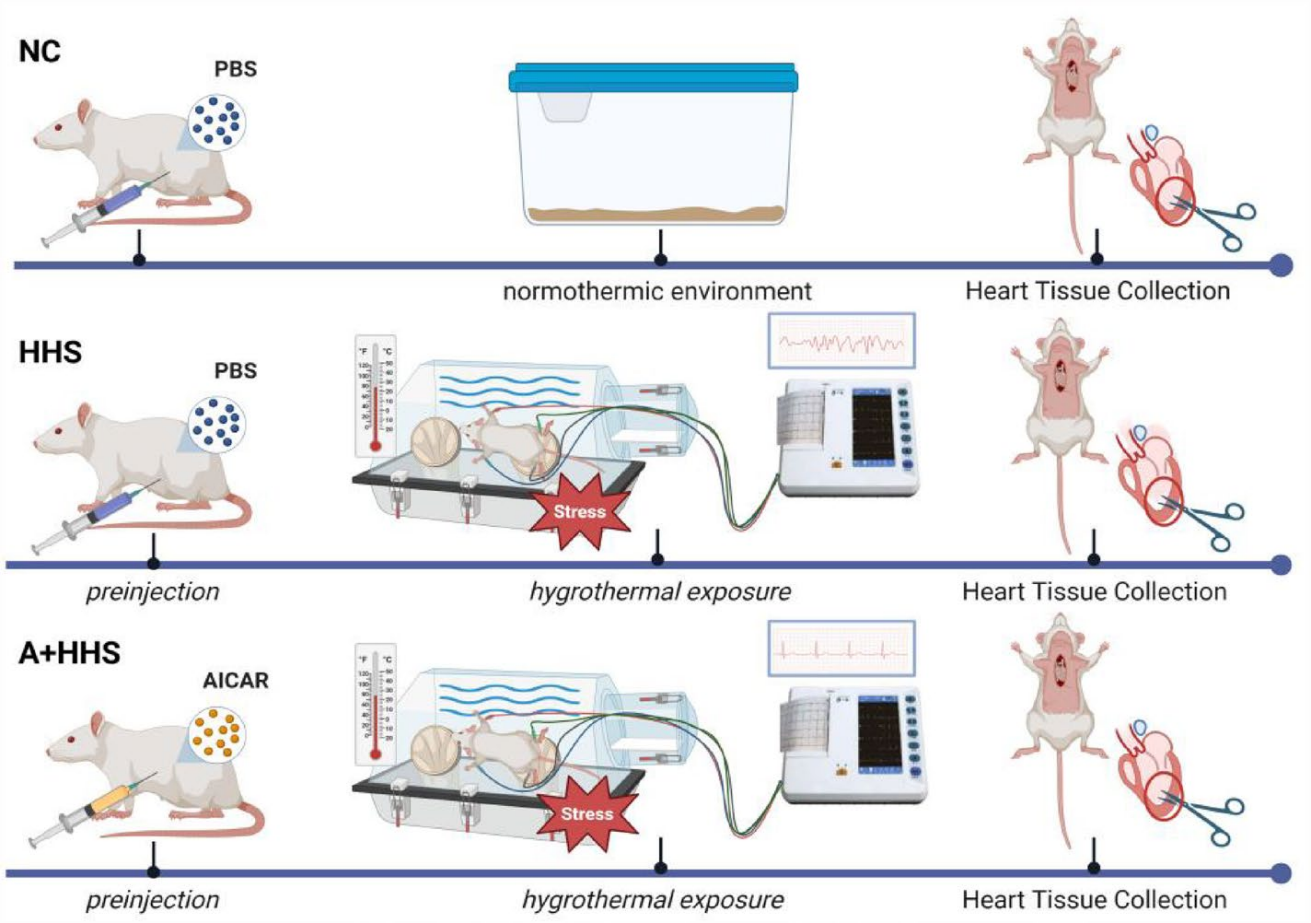
The molding process diagram. Rats categorized as NC, HHS, and A + HHS groups received pretreatment with either 1 ml of PBS or AICAR. Subsequently, the HHS and A + HHS groups were subjected to stress exposure within a simulation chamber, while real-time monitoring of arrhythmia was conducted using an electrocardiogram machine. At the conclusion of the modeling, blood was collected from the heart, and left ventricular myocardial tissues were isolated for subsequent experimental analysis.

24 SD rats were randomly divided into one of the following three groups of eight rats each: NC (normothermic controls) group: rats pretreated with 1ml PBS by intraperitoneal injection and kept under standard experimental conditions throughout the entire experiment; HHS group: rats pretreated with 1ml PBS by intraperitoneal injection and received HHS; and Acadesine, AICAR (A) + HHS group (with AMPK agonist): rats were pretreated with AICAR (A8184, APExBIO), dissolved in 0.1% DMSO in phosphate buffered saline (PBS), and administered in a dose of 500 mg/kg by intraperitoneal injection 1 h prior to the start of HHS (Fig. 7). Then, the two groups, HHS and A + HHS, were placed in a simulation chamber for stress exposure and real-time ECG monitoring was performed to record the heart rate and the occurrence of arrhythmic events. After the end of modeling, blood samples and left ventricular (LV) tissue were taken for biochemical analysis and histology.

### Basic physiological parameters measure

Rat rectal temperature (Tr) was measured using an animal-specific electronic thermometer (OMRON Co., Ltd., Dalian), and Tr represented core body temperature. Tr was recorded for seven days, and the basic core temperature (Tcb) was calculated using the mean Tr. Additionally, baseline weight was assessed and recorded before each group of rats undergoing HHS, and again following the conclusion of modeling. Meanwhile, rats in each group were anesthetized by intraperitoneal injection of sodium pentobarbital (30 mg/kg) 1 day before the experiment, fixed in the supine position, and then the limbs were dehairing and connected to the veterinary electrocardiogram machine (HB-A3, Zhuhai Hongbang Medical Technology Co., Ltd.) to record the basic electrocardiographic parameters, such as the base heart rate (BHR) and the standard limb-lead electrocardiographic waveforms (Table 1).

**Table 1:** Basic physiological parameters.

Parameters	NC (n = 8)	HHS (n = 8)	A + HHS (n = 8)	*P*-value
Weight (g)	305 ± 16.65	312.4 ± 14.26	308 ± 19.86	0.690
Tcb (℃)	36.9 ± 0.15	37.0 ± 0.21	36.9 ± 0.19	0.564
BHR, beats (min)	346.4 ± 39.81	350.3 ± 38.12	348.9 ± 32.17	0.978

There were no statistically significant differences in preexperimental baseline levels of body weight, Tcb, and BHR among the three groups. Tcb, basic core temperature; BHR, base heart rate.

### Hygrothermal exposure

According to the studied literature^38–40^, the experimental rats were maintained normally at the hospital laboratory animal facility for a week to acclimate to the environment and fasted for 12 h before the stimulation. Prior to the hygrothermal stimulation, the artificial thermal climate simulation chamber (920 incubator, China Ningbo David medical equipment co., LTD.) was preheated to 40 °C with 85% humidity, and then, except for the NC group, which was placed in a room temperature environment throughout the whole process, the rats in the HHS and the A + HHS group were put into the chamber in batches for the stimulation to prepare the HHS model. Tr was monitored transrectally throughout the modeling period (probe implanted into the anus at about 3–5 cm) and recorded every 10 min until Tr reached the (42.5 ± 0.5) °C modeling success threshold, as well as the duration of any arrhythmic events that occurred during HHS.

### ECG acquisition and analysis

As shown in Fig. 7, rat limbs were linked to the ECG machine during HHS, and the self-contained ECG module examined the ECG parameters. Arrhythmic events and changes in HR were recorded (feed speed, 25 mm/s, voltage, 10 mV).

### ELISA

After cardiac monitoring, blood from the heart was drawn into sterile tubes and centrifuged at 2000 rpm for 15 min at room temperature in order to separate the supernatant, which was then stored in the refrigerator at − 80 °C to measure the level of serum stress hormone indicators CRH, ACTH, CORT and inflammatory factors TNF-α, IL-1β. On the day of the experiment, the ELISA kits (EK11288, EK12421, EK11067, EK1940, EK17664, SAB, America) were rewarmed at room temperature, followed the instructions, and measured the absorbance value (OD) of each well by enzyme meter (Thermo, Multiskan Go S/N: 1510-00633, Thermo Fisher technology company). The concentration and OD value of the standard was used as a reference to compute the concentration of the sample by drawing the standard curve with CurveExpert software.

### Western blot

First, myocardial tissue proteins were extracted by adding 10 μL of lysis buffer (Protease Inhibitor Mix, Phosphatase Inhibitor Mix, EDTA, and PMSF in a proportional ratio) to each 1 mg of frozen LV tissue, then lysed on ice for 30 min and centrifuged at 12,000 rpm/min at 4 °C for 15 min. Then, the BCA protein assay kit (Beyotime, No. P0010, Shanghai, China) was used to measure the total proteins in the cardiac tissues. Equal amounts of proteins per lane were separated in 10% SDS–polyacrylamide gels at 120 V (PG112, EpiZyme, China). Then, the appropriate size 0.45 μm PVDF membrane were cut, labeled and immersed in methanol for a few seconds for activation, and the gel were transferred to PVDF membranes (IPVH00010, Millipore, American) and blocked with a Rapid Block (Beyotime, P0235, Shanghai) for 30 min, followed by incubation overnight at 4 °C with the following primary antibodies, anti-ser368-phosphorylated Cx43 (1:1000, 3511S, CST), anti-total Cx43 (1:1000, 3512S, CST), anti-thr172-phosphorylated AMPK (1:1000, 2535T, CST), anti-total AMPK (1:1000, 2532S, CST), anti-lkb1 (1:1000, 3482S, CST), and anti-GAPDH (1:5000, ab9485, Abcam). After that, the membranes were gently washed using Tris-buffered 0.1% Tween (TBS-T) and subsequently incubated for 1 h with a secondary antibody linked to horseradish peroxidase (goat anti-rabbit, 1:3000, ab205718, Abcam, America) and washed in TBS-T. Finally, the results were visualized using ECL luminous solution (WBKLS0100, Merck Millipore, America). To view the images, chemiluminescence imaging equipment (Guangyi Biotechnology Co., Ltd., Guangzhou, China) was employed. Image J software (version 1.52v, the National Institutes of Health in Rasband, USA) was applied for analysis, and the target protein bands were normalized by the gray value of the bands of the internal reference GAPDH.

### H&E and Masson maintenance staining

The LV tissues preserved in 4% paraformaldehyde were embedded in paraffin and serially sectioned to a thickness of 4 µm. The tissue sections underwent a meticulous processing protocol, starting with incubation in an oven at 60 °C for 20 min, followed by dual immersion in xylene for dewaxing. Subsequently, the sections underwent rehydration through a series of graded ethanol concentrations, then immersion in hematoxylin for staining. Differentiation was achieved using 1% hydrochloric acid in ethanol, and a return to blue was facilitated with ammonia. Afterward, the sections were immersed and stained with alcohol-soluble eosin, subjected to dehydration in graded ethanol concentrations, and underwent triple immersion in xylene for transparency. The final step involved sealing the slices with neutral resin, allowing for pathological examination of myocardial tissue changes under microscope. In addition, we used a modified Masson trichrome staining kit (G1346, Solarbio, China) for myocardial fibrosis detection. The dewaxing and rehydration steps are the same as those described for H&E. After that, stained with Mayer's hematoxylin staining solution and then differentiated in acid-alcohol differentiation solution, stained with cochineal staining solution and rinsed with distilled water, then treated with phosphomolybdic acid hydrate solution and stained with aniline blue staining solution. Finally, the films were treated with a weak acid solution, then quickly dehydrated with ethanol, transparency with xylene, and sealed with a neutral resin so that myocardial fibrotic changes could be observed under the microscope. Both H&E and Masson staining results were observed using thea BX-51 fluorescent digital imaging microscope (Olympus, Japan).

### Immunohistochemistry (IHC)

First, 4µm serial paraffin sections of LV myocardial tissue were taken. Subsequently, the sections were baked in an oven at 60 °C for 2 h, dewaxed, rehydrated, and treated with a 3% H_2_O_2_ solution for 5–10 min at room temperature. After rinsing in PBS buffer, the sections were immersed in sodium citrate antigen repair solution for thermal repair for 5 min, followed by cooling to room temperature and then 5% BSA Block was added at 37 °C for 30 min. After that, anti-Cx43-pS368 antibody was added dropwise at 4 °C overnight, followed by rinsing and then incubated at 37 °C for 30 min with HRP-linked anti-rabbit secondary antibody. Finally, an appropriate amount of DAB Staining Sloution (AR1027, Boster, China) was added dropwise. Microscope control was employed to monitor the reaction time. Afterwards, the sections were restained with hematoxylin, underwent 1% hydrochloric acid–ethanol differentiation, and were subjected to dehydration, transparency, and sealing steps. The expression and distribution of Cx43 was observed under the BX-51 microscope, and the images were analyzed by Image J.

### Transmission electron microscopy (TEM)

Left ventricular heart tissues (1 mm * 1 mm * 1 mm) were removed immediately after modeling was complete, placed in 2.5% glutaraldehyde fixative at 4 °C, fixed with osmium tetroxide solution for 2 h at room temperature, dehydrated, replaced, baked at 60 °C for 48 h, and cryo-microtomized at 40–60 nm on a microtomography machine (EM UC7, Leica), and slices were fished with a copper mesh. TEM (HT7700, Hitachi, Japan) was carried out at 2500 × to 10,000 × magnificationswas carried out at 2500 × to 10,000 × magnifications to evaluate the microstructural alterations such as gap junctions and mitochondria in stained sections after they had been double-stained with saturated uranyl acetate and lead citrate.

### Statistical methods

To compare the three groups, a one-way ANOVA was performed using GraphPad Prism, version 8.0 (GraphPad Software Inc., San Diego, CA, USA). Image J, version 1.52v, from the National Institutes of Health in Rasband, USA, was used to analyze the outcomes of the WB, IHC, and Masson tests. All quantitative data were presented as mean standard deviation (x ± SD). Statistical significance was defined as a value of *p* < 0.05.

### Supplementary Information

Supplementary Information.

## Data Availability

The datasets generated during and/or analysed during the current study are available from the corresponding author on reasonable request.
