# Supplementary material for: Hygrothermal stress increases malignant arrhythmias susceptibility by inhibiting the LKB1-AMPK-Cx43 pathway
Source: Sci Rep. 2024 Feb 29;14:5010. doi: 10.1038/s41598-024-55804-0 (PMC10904738; doi:10.1038/s41598-024-55804-0)

Figure 4A.a p-Cx43

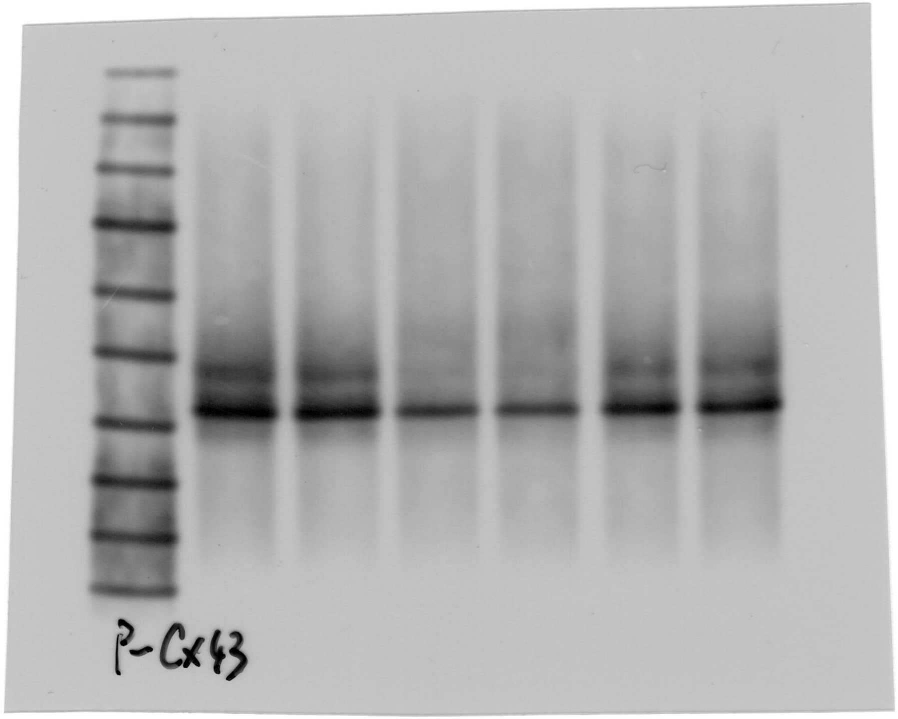

Figure 4A.a t-Cx43

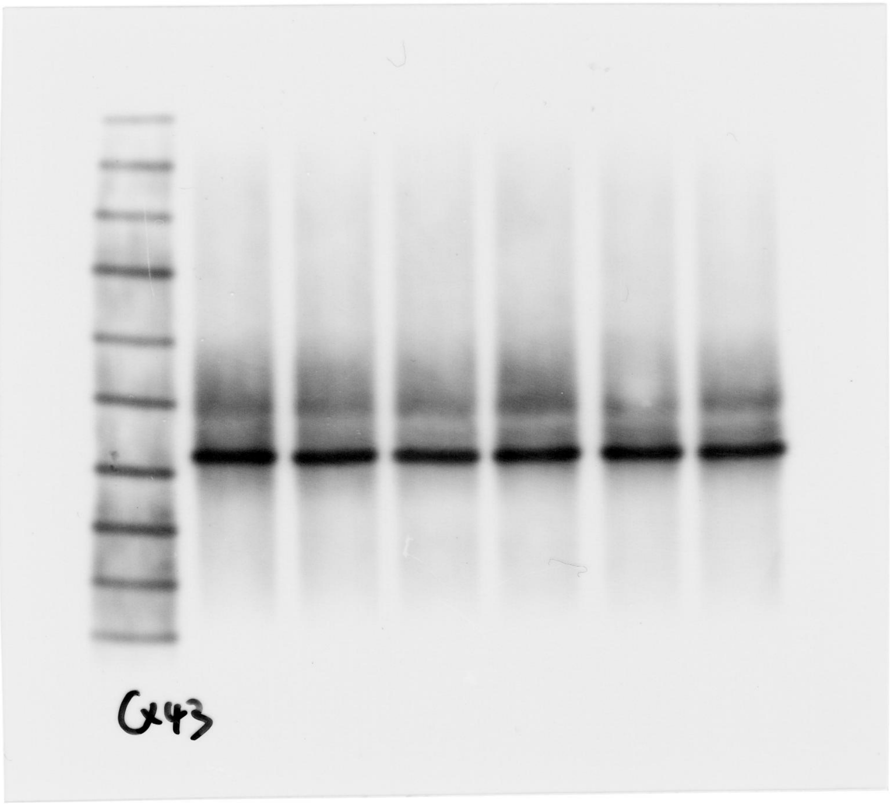

Figure 4A.a GAPDH

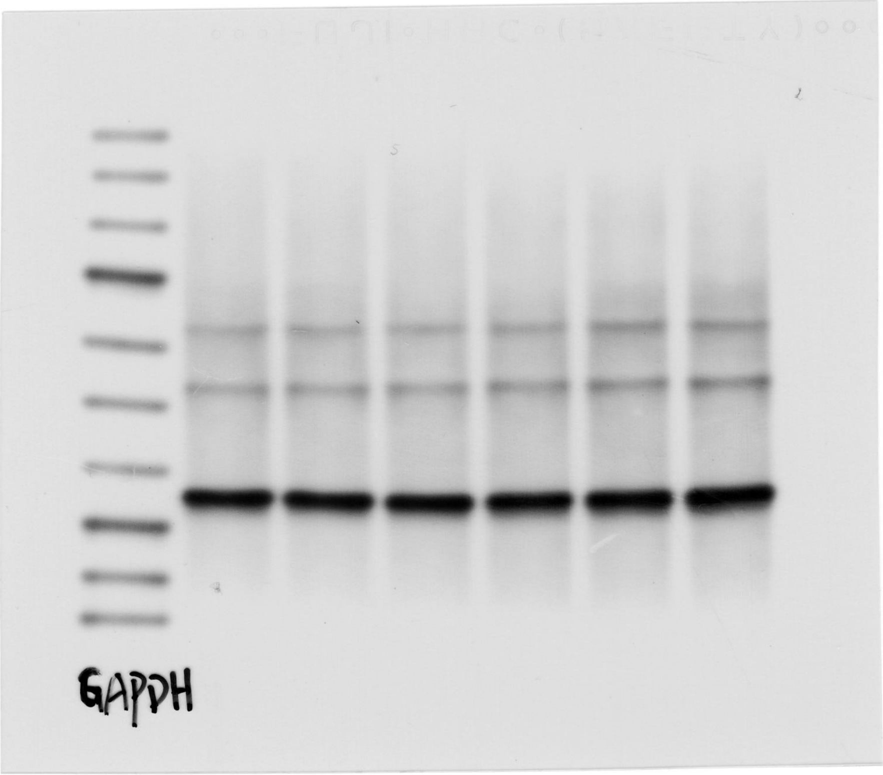

Figure 4A.a p-AMPK

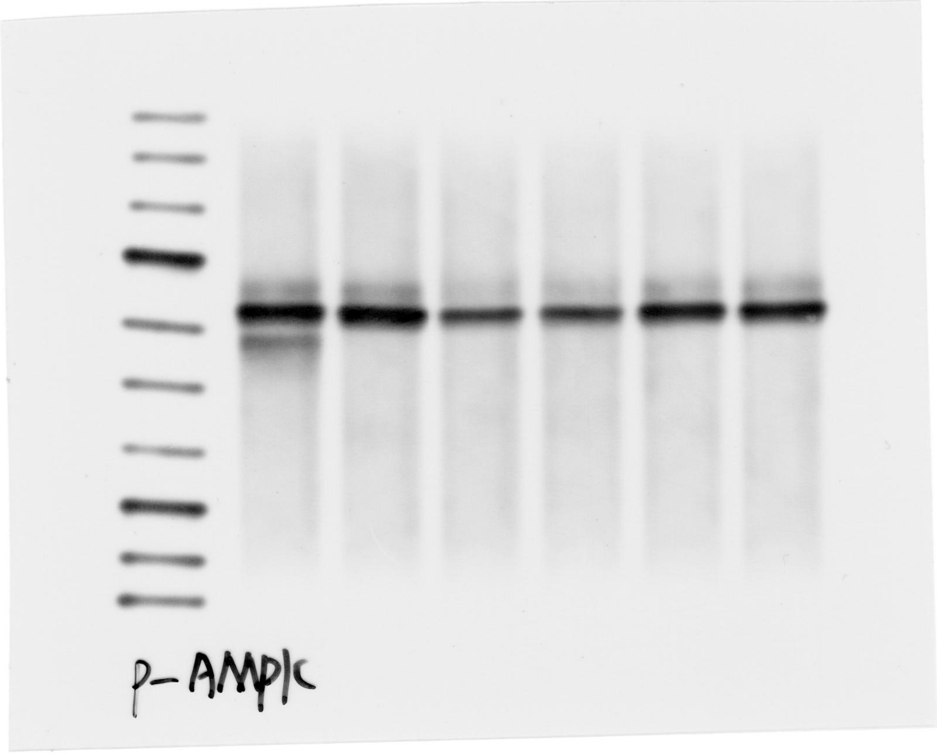

Figure 4A.a AMPK

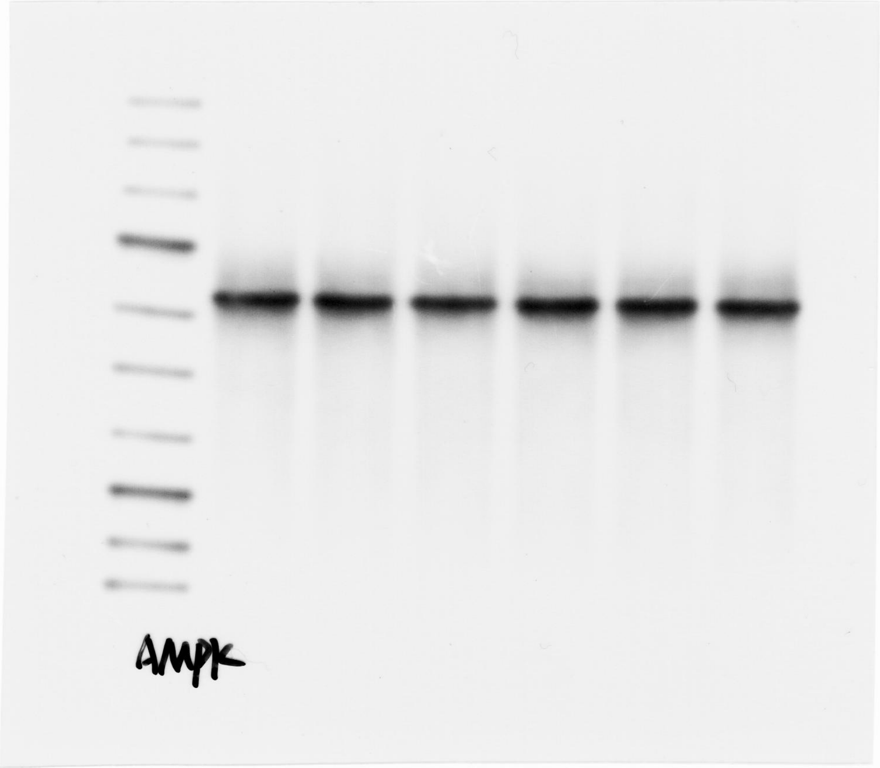

Figure 4A.a LKB1

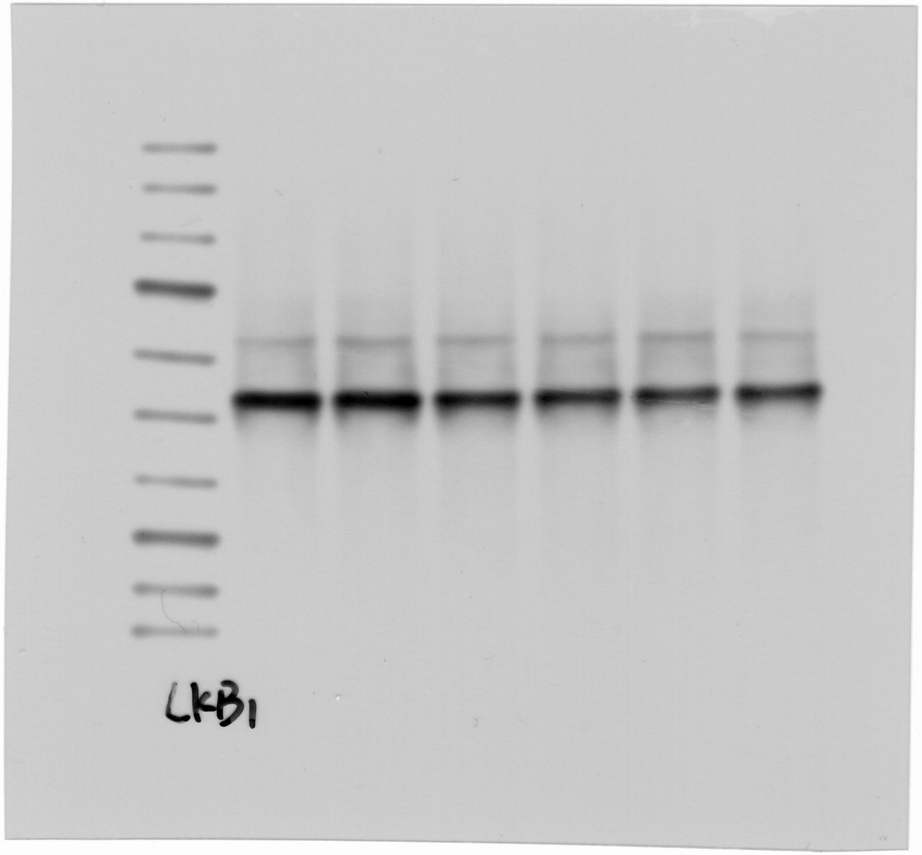

Figure 4A.a GAPDH

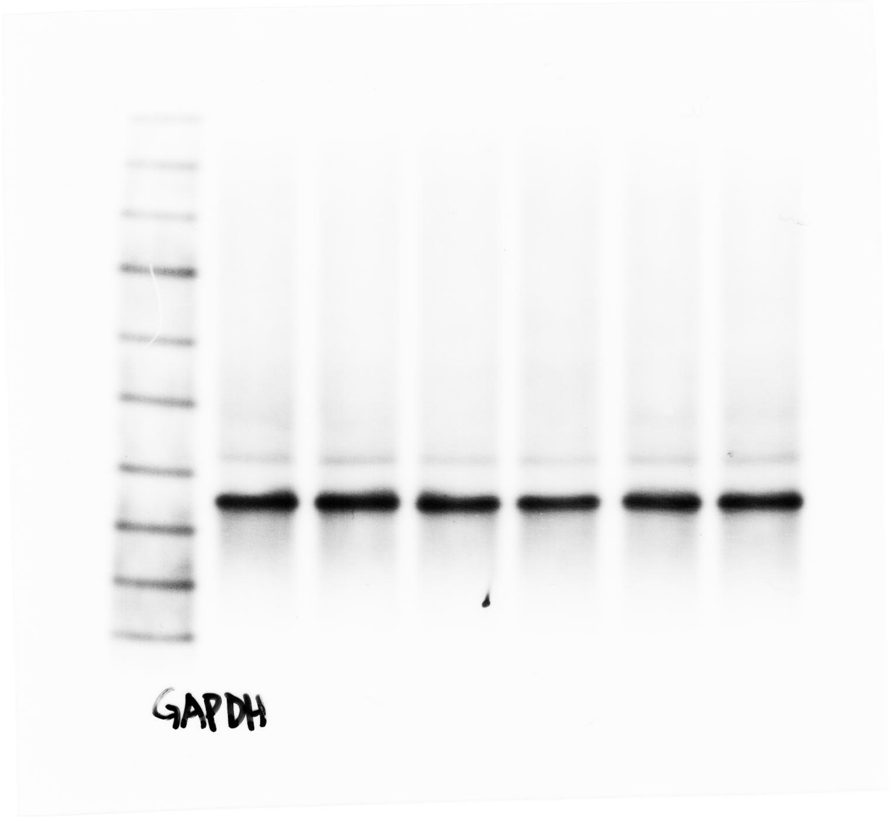

Figure 5D.a ST2

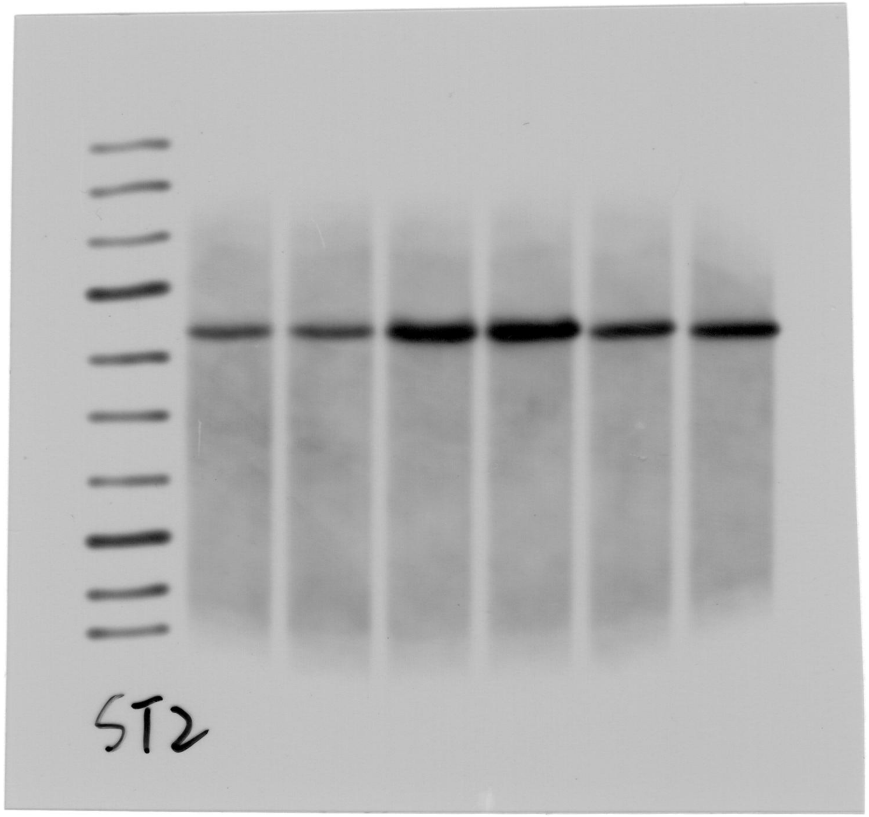

Figure 5D.a GAPDH

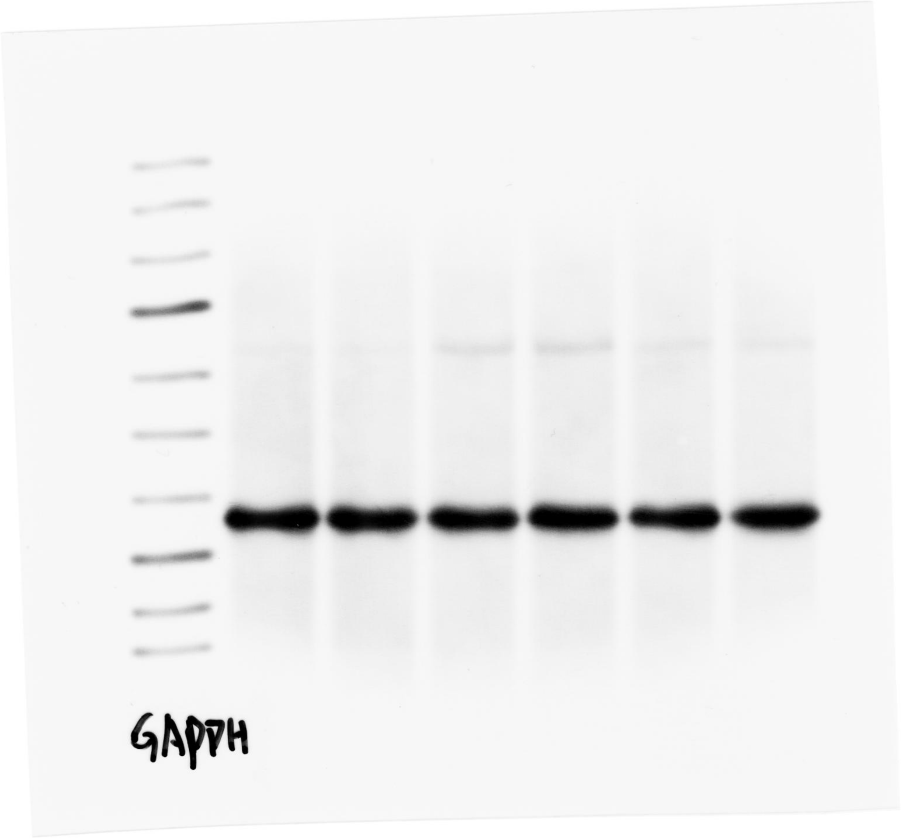

Supplement: Supplementary file 1 — Supplementary Information. [file 41598_2024_55804_MOESM1_ESM.pdf]
